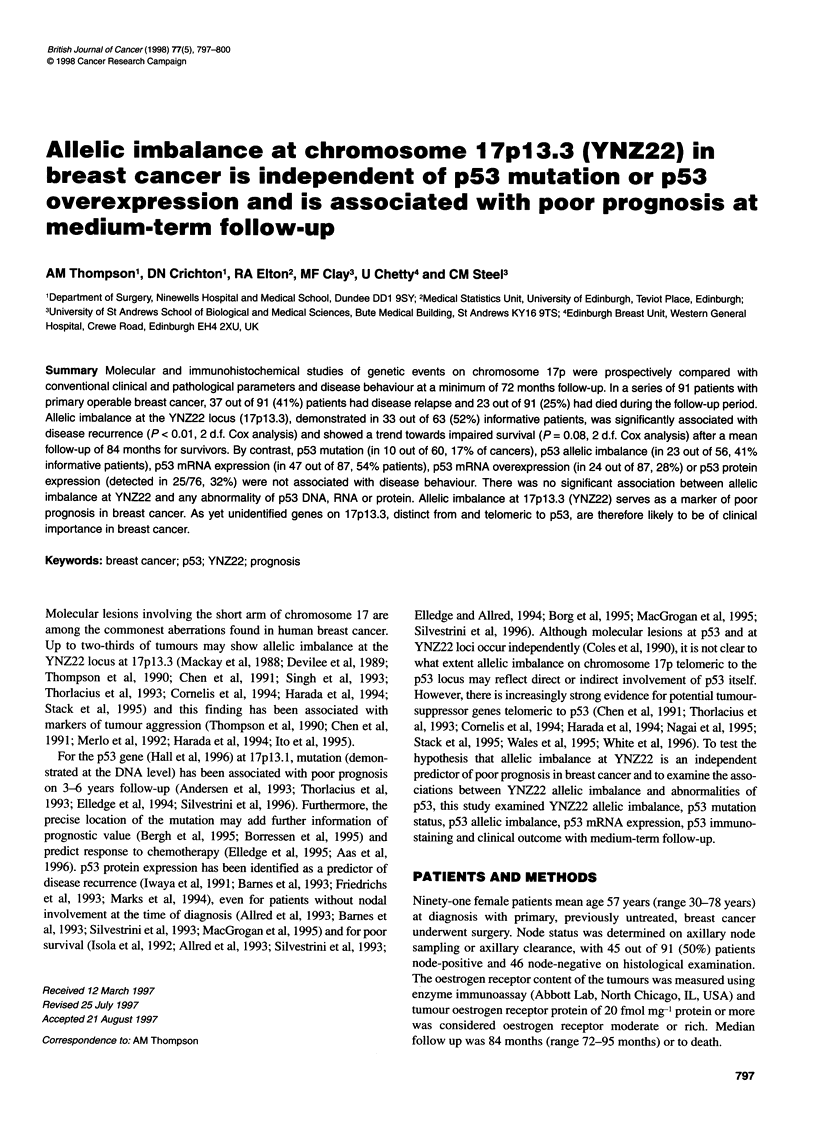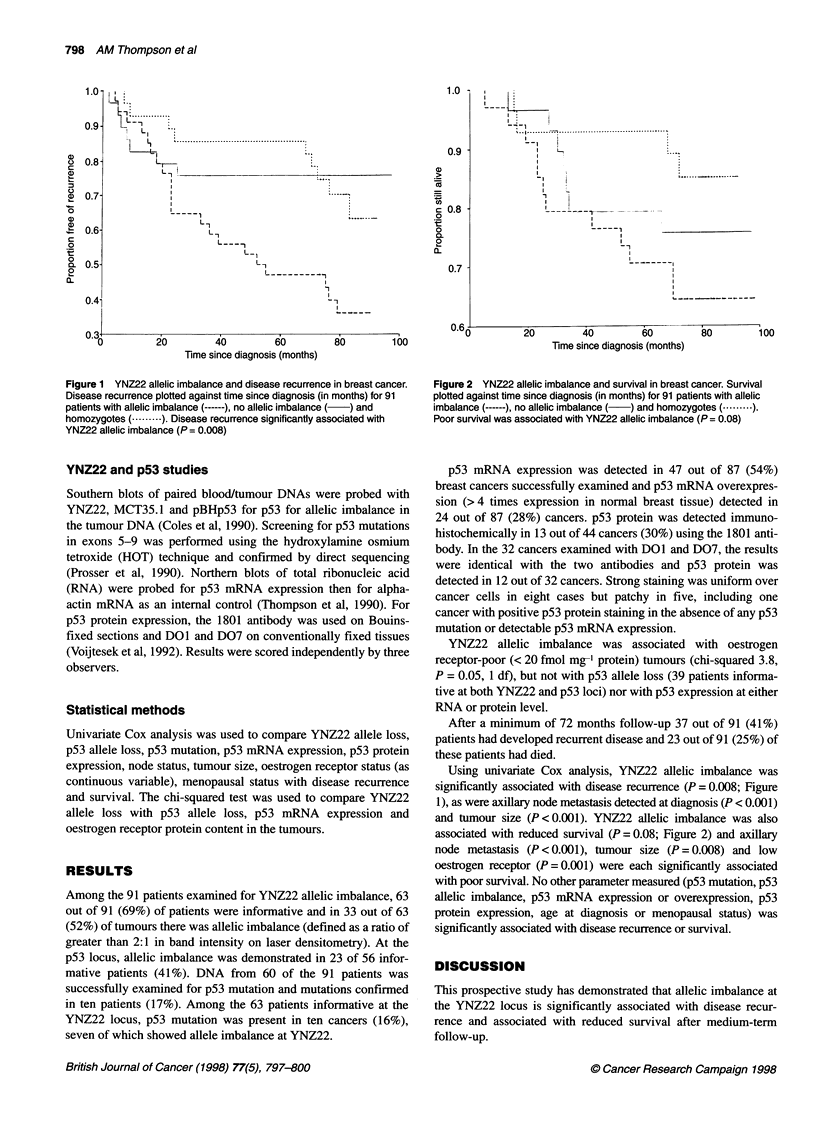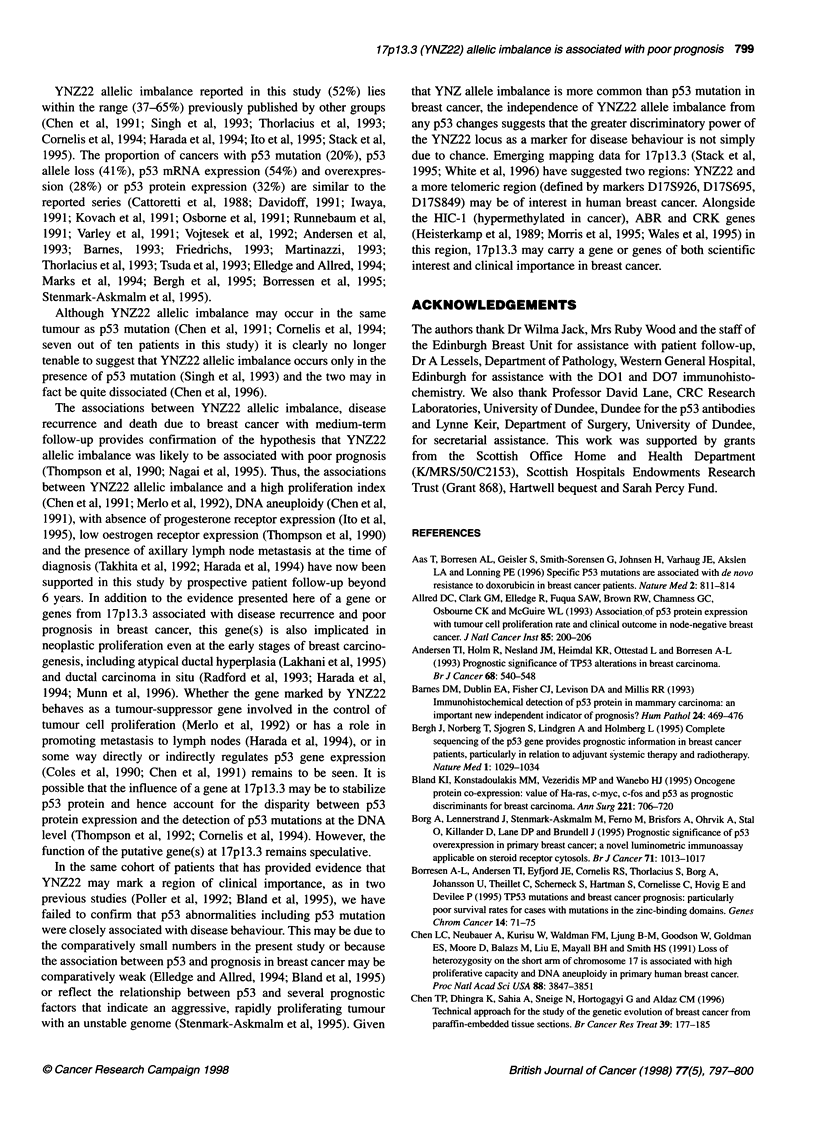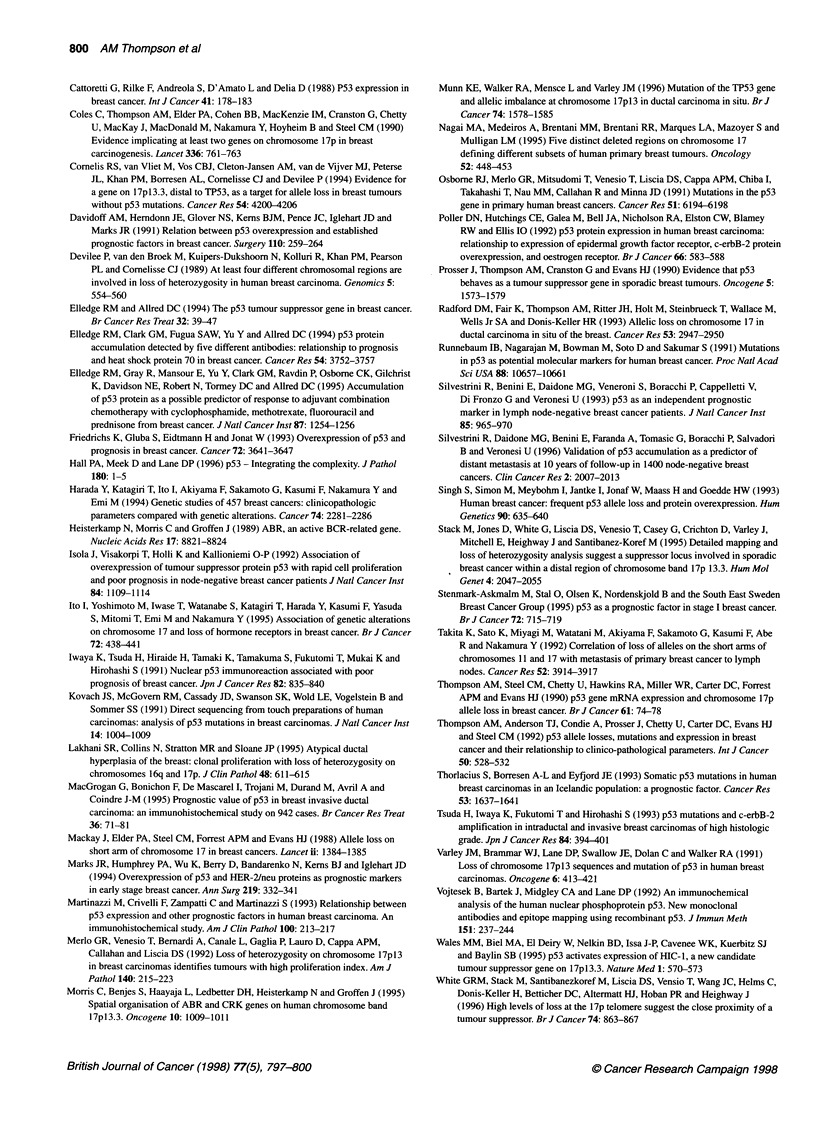# Allelic imbalance at chromosome 17p13.3 (YNZ22) in breast cancer is independent of p53 mutation or p53 overexpression and is associated with poor prognosis at medium-term follow-up.

**DOI:** 10.1038/bjc.1998.129

**Published:** 1998-03

**Authors:** A. M. Thompson, D. N. Crichton, R. A. Elton, M. F. Clay, U. Chetty, C. M. Steel

**Affiliations:** Department of Surgery, Ninewells Hospital and Medical School, Dundee, UK.

## Abstract

Molecular and immunohistochemical studies of genetic events on chromosome 17p were prospectively compared with conventional clinical and pathological parameters and disease behaviour at a minimum of 72 months follow-up. In a series of 91 patients with primary operable breast cancer, 37 out of 91 (41%) patients had disease relapse and 23 out of 91 (25%) had died during the follow-up period. Allelic imbalance at the YNZ22 locus (17p13.3), demonstrated in 33 out of 63 (52%) informative patients, was significantly associated with disease recurrence (P < 0.01, 2 d.f. Cox analysis) and showed a trend towards impaired survival (P = 0.08, 2 d.f. Cox analysis) after a mean follow-up of 84 months for survivors. By contrast, p53 mutation (in 10 out of 60, 17% of cancers), p53 allelic imbalance (in 23 out of 56, 41% informative patients), p53 mRNA expression (in 47 out of 87, 54% patients), p53 mRNA overexpression (in 24 out of 87, 28%) or p53 protein expression (detected in 25/76, 32%) were not associated with disease behaviour. There was no significant association between allelic imbalance at YNZ22 and any abnormality of p53 DNA, RNA or protein. Allelic imbalance at 17p13.3 (YNZ22) serves as a marker of poor prognosis in breast cancer. As yet unidentified genes on 17p13.3, distinct from and telomeric to p53, are therefore likely to be of clinical importance in breast cancer.


British Journal of Cancer (1998) 77(5), 797–800
© 1998 Cancer Research Campaign

### Allelic imbalance at chromosome 17p13.3 (YNZ22) in breast cancer is independent of p53 mutation or p53 overexpression and is associated with poor prognosis at medium-term follow-up

**AM Thompson[1], DN Crichton[1], RA Elton[2], MF Clay[3], U Chetty[4] and CM Steel[3]**

[1]Department of Surgery, Ninewells Hospital and Medical School, Dundee DD1 9SY; [2]Medical Statistics Unit, University of Edinburgh, Teviot Place, Edinburgh; [3]University of St Andrews School of Biological and Medical Sciences, Bute Medical Building, St Andrews KY16 9TS; [4]Edinburgh Breast Unit, Western General Hospital, Crewe Road, Edinburgh EH4 2XU, UK

**Summary** Molecular and immunohistochemical studies of genetic events on chromosome 17p were prospectively compared with conventional clinical and pathological parameters and disease behaviour at a minimum of 72 months follow-up. In a series of 91 patients with primary operable breast cancer, 37 out of 91 (41%) patients had disease relapse and 23 out of 91 (25%) had died during the follow-up period. Allelic imbalance at the YNZ22 locus (17p13.3), demonstrated in 33 out of 63 (52%) informative patients, was significantly associated with disease recurrence ($P < 0.01$, 2 d.f. Cox analysis) and showed a trend towards impaired survival ($P = 0.08$, 2 d.f. Cox analysis) after a mean follow-up of 84 months for survivors. By contrast, p53 mutation (in 10 out of 60, 17% of cancers), p53 allelic imbalance (in 23 out of 56, 41% informative patients), p53 mRNA expression (in 47 out of 87, 54% patients), p53 mRNA overexpression (in 24 out of 87, 28%) or p53 protein expression (detected in 25/76, 32%) were not associated with disease behaviour. There was no significant association between allelic imbalance at YNZ22 and any abnormality of p53 DNA, RNA or protein. Allelic imbalance at 17p13.3 (YNZ22) serves as a marker of poor prognosis in breast cancer. As yet unidentified genes on 17p13.3, distinct from and telomeric to p53, are therefore likely to be of clinical importance in breast cancer.

**Keywords:** breast cancer; p53; YNZ22; prognosis

Molecular lesions involving the short arm of chromosome 17 are among the commonest aberrations found in human breast cancer. Up to two-thirds of tumours may show allelic imbalance at the YNZ22 locus at 17p13.3 (Mackay et al, 1988; Devilee et al, 1989; Thompson et al, 1990; Chen et al, 1991; Singh et al, 1993; Thorlacius et al, 1993; Cornelis et al, 1994; Harada et al, 1994; Stack et al, 1995) and this finding has been associated with markers of tumour aggression (Thompson et al, 1990; Chen et al, 1991; Merlo et al, 1992; Harada et al, 1994; Ito et al, 1995).

For the p53 gene (Hall et al, 1996) at 17p13.1, mutation (demonstrated at the DNA level) has been associated with poor prognosis on 3–6 years follow-up (Andersen et al, 1993; Thorlacius et al, 1993; Elledge et al, 1994; Silvestrini et al, 1996). Furthermore, the precise location of the mutation may add further information of prognostic value (Bergh et al, 1995; Borressen et al, 1995) and predict response to chemotherapy (Elledge et al, 1995; Aas et al, 1996). p53 protein expression has been identified as a predictor of disease recurrence (Iwaya et al, 1991; Barnes et al, 1993; Friedrichs et al, 1993; Marks et al, 1994), even for patients without nodal involvement at the time of diagnosis (Allred et al, 1993; Barnes et al, 1993; Silvestrini et al, 1993; MacGrogan et al, 1995) and for poor survival (Isola et al, 1992; Allred et al, 1993; Silvestrini et al, 1993; Elledge and Allred, 1994; Borg et al, 1995; MacGrogan et al, 1995; Silvestrini et al, 1996). Although molecular lesions at p53 and at YNZ22 loci occur independently (Coles et al, 1990), it is not clear to what extent allelic imbalance on chromosome 17p telomeric to the p53 locus may reflect direct or indirect involvement of p53 itself. However, there is increasingly strong evidence for potential tumour-suppressor genes telomeric to p53 (Chen et al, 1991; Thorlacius et al, 1993; Cornelis et al, 1994; Harada et al, 1994; Nagai et al, 1995; Stack et al, 1995; Wales et al, 1995; White et al, 1996). To test the hypothesis that allelic imbalance at YNZ22 is an independent predictor of poor prognosis in breast cancer and to examine the associations between YNZ22 allelic imbalance and abnormalities of p53, this study examined YNZ22 allelic imbalance, p53 mutation status, p53 allelic imbalance, p53 mRNA expression, p53 immunostaining and clinical outcome with medium-term follow-up.

*Received 12 March 1997*
*Revised 25 July 1997*
*Accepted 21 August 1997*

*Correspondence to:* AM Thompson

### PATIENTS AND METHODS

Ninety-one female patients mean age 57 years (range 30–78 years) at diagnosis with primary, previously untreated, breast cancer underwent surgery. Node status was determined on axillary node sampling or axillary clearance, with 45 out of 91 (50%) patients node-positive and 46 node-negative on histological examination. The oestrogen receptor content of the tumours was measured using enzyme immunoassay (Abbott Lab, North Chicago, IL, USA) and tumour oestrogen receptor protein of 20 fmol mg$^{-1}$ protein or more was considered oestrogen receptor moderate or rich. Median follow up was 84 months (range 72–95 months) or to death.

**Figure 1** YNZ22 allelic imbalance and disease recurrence in breast cancer. Disease recurrence plotted against time since diagnosis (in months) for 91 patients with allelic imbalance (------), no allelic imbalance (——) and homozygotes (·········). Disease recurrence significantly associated with YNZ22 allelic imbalance ($P = 0.008$)

**Figure 2** YNZ22 allelic imbalance and survival in breast cancer. Survival plotted against time since diagnosis (in months) for 91 patients with allelic imbalance (------), no allelic imbalance (——) and homozygotes (·········). Poor survival was associated with YNZ22 allelic imbalance ($P = 0.08$)

### YNZ22 and p53 studies

Southern blots of paired blood/tumour DNAs were probed with YNZ22, MCT35.1 and pBHp53 for p53 for allelic imbalance in the tumour DNA (Coles et al, 1990). Screening for p53 mutations in exons 5–9 was performed using the hydroxylamine osmium tetroxide (HOT) technique and confirmed by direct sequencing (Prosser et al, 1990). Northern blots of total ribonucleic acid (RNA) were probed for p53 mRNA expression then for alpha-actin mRNA as an internal control (Thompson et al, 1990). For p53 protein expression, the 1801 antibody was used on Bouins-fixed sections and DO1 and DO7 on conventionally fixed tissues (Voijtesek et al, 1992). Results were scored independently by three observers.

### Statistical methods

Univariate Cox analysis was used to compare YNZ22 allele loss, p53 allele loss, p53 mutation, p53 mRNA expression, p53 protein expression, node status, tumour size, oestrogen receptor status (as continuous variable), menopausal status with disease recurrence and survival. The chi-squared test was used to compare YNZ22 allele loss with p53 allele loss, p53 mRNA expression and oestrogen receptor protein content in the tumours.

### RESULTS

Among the 91 patients examined for YNZ22 allelic imbalance, 63 out of 91 (69%) of patients were informative and in 33 out of 63 (52%) of tumours there was allelic imbalance (defined as a ratio of greater than 2:1 in band intensity on laser densitometry). At the p53 locus, allelic imbalance was demonstrated in 23 of 56 informative patients (41%). DNA from 60 of the 91 patients was successfully examined for p53 mutation and mutations confirmed in ten patients (17%). Among the 63 patients informative at the YNZ22 locus, p53 mutation was present in ten cancers (16%), seven of which showed allele imbalance at YNZ22.

p53 mRNA expression was detected in 47 out of 87 (54%) breast cancers successfully examined and p53 mRNA overexpression (> 4 times expression in normal breast tissue) detected in 24 out of 87 (28%) cancers. p53 protein was detected immunohistochemically in 13 out of 44 cancers (30%) using the 1801 antibody. In the 32 cancers examined with DO1 and DO7, the results were identical with the two antibodies and p53 protein was detected in 12 out of 32 cancers. Strong staining was uniform over cancer cells in eight cases but patchy in five, including one cancer with positive p53 protein staining in the absence of any p53 mutation or detectable p53 mRNA expression.

YNZ22 allelic imbalance was associated with oestrogen receptor-poor (< 20 fmol $mg^{-1}$ protein) tumours (chi-squared 3.8, $P = 0.05$, 1 df), but not with p53 allele loss (39 patients informative at both YNZ22 and p53 loci) nor with p53 expression at either RNA or protein level.

After a minimum of 72 months follow-up 37 out of 91 (41%) patients had developed recurrent disease and 23 out of 91 (25%) of these patients had died.

Using univariate Cox analysis, YNZ22 allelic imbalance was significantly associated with disease recurrence ($P = 0.008$; Figure 1), as were axillary node metastasis detected at diagnosis ($P < 0.001$) and tumour size ($P < 0.001$). YNZ22 allelic imbalance was also associated with reduced survival ($P = 0.08$; Figure 2) and axillary node metastasis ($P < 0.001$), tumour size ($P = 0.008$) and low oestrogen receptor ($P = 0.001$) were each significantly associated with poor survival. No other parameter measured (p53 mutation, p53 allelic imbalance, p53 mRNA expression or overexpression, p53 protein expression, age at diagnosis or menopausal status) was significantly associated with disease recurrence or survival.

### DISCUSSION

This prospective study has demonstrated that allelic imbalance at the YNZ22 locus is significantly associated with disease recurrence and associated with reduced survival after medium-term follow-up.

© Cancer Research Campaign 1998

YNZ22 allelic imbalance reported in this study (52%) lies within the range (37–65%) previously published by other groups (Chen et al, 1991; Singh et al, 1993; Thorlacius et al, 1993; Cornelis et al, 1994; Harada et al, 1994; Ito et al, 1995; Stack et al, 1995). The proportion of cancers with p53 mutation (20%), p53 allele loss (41%), p53 mRNA expression (54%) and overexpression (28%) or p53 protein expression (32%) are similar to the reported series (Cattoretti et al, 1988; Davidoff, 1991; Iwaya, 1991; Kovach et al, 1991; Osborne et al, 1991; Runnebaum et al, 1991; Varley et al, 1991; Vojtesek et al, 1992; Andersen et al, 1993; Barnes, 1993; Friedrichs, 1993; Martinazzi, 1993; Thorlacius et al, 1993; Tsuda et al, 1993; Elledge and Allred, 1994; Marks et al, 1994; Bergh et al, 1995; Borresen et al, 1995; Stenmark-Askmalm et al, 1995).

Although YNZ22 allelic imbalance may occur in the same tumour as p53 mutation (Chen et al, 1991; Cornelis et al, 1994; seven out of ten patients in this study) it is clearly no longer tenable to suggest that YNZ22 allelic imbalance occurs only in the presence of p53 mutation (Singh et al, 1993) and the two may in fact be quite dissociated (Chen et al, 1996).

The associations between YNZ22 allelic imbalance, disease recurrence and death due to breast cancer with medium-term follow-up provides confirmation of the hypothesis that YNZ22 allelic imbalance was likely to be associated with poor prognosis (Thompson et al, 1990; Nagai et al, 1995). Thus, the associations between YNZ22 allelic imbalance and a high proliferation index (Chen et al, 1991; Merlo et al, 1992), DNA aneuploidy (Chen et al, 1991), with absence of progesterone receptor expression (Ito et al, 1995), low oestrogen receptor expression (Thompson et al, 1990) and the presence of axillary lymph node metastasis at the time of diagnosis (Takhita et al, 1992; Harada et al, 1994) have now been supported in this study by prospective patient follow-up beyond 6 years. In addition to the evidence presented here of a gene or genes from 17p13.3 associated with disease recurrence and poor prognosis in breast cancer, this gene(s) is also implicated in neoplastic proliferation even at the early stages of breast carcinogenesis, including atypical ductal hyperplasia (Lakhani et al, 1995) and ductal carcinoma in situ (Radford et al, 1993; Harada et al, 1994; Munn et al, 1996). Whether the gene marked by YNZ22 behaves as a tumour-suppressor gene involved in the control of tumour cell proliferation (Merlo et al, 1992) or has a role in promoting metastasis to lymph nodes (Harada et al, 1994), or in some way directly or indirectly regulates p53 gene expression (Coles et al, 1990; Chen et al, 1991) remains to be seen. It is possible that the influence of a gene at 17p13.3 may be to stabilize p53 protein and hence account for the disparity between p53 protein expression and the detection of p53 mutations at the DNA level (Thompson et al, 1992; Cornelis et al, 1994). However, the function of the putative gene(s) at 17p13.3 remains speculative.

In the same cohort of patients that has provided evidence that YNZ22 may mark a region of clinical importance, as in two previous studies (Poller et al, 1992; Bland et al, 1995), we have failed to confirm that p53 abnormalities including p53 mutation were closely associated with disease behaviour. This may be due to the comparatively small numbers in the present study or because the association between p53 and prognosis in breast cancer may be comparatively weak (Elledge and Allred, 1994; Bland et al, 1995) or reflect the relationship between p53 and several prognostic factors that indicate an aggressive, rapidly proliferating tumour with an unstable genome (Stenmark-Askmalm et al, 1995). Given that YNZ allele imbalance is more common than p53 mutation in breast cancer, the independence of YNZ22 allele imbalance from any p53 changes suggests that the greater discriminatory power of the YNZ22 locus as a marker for disease behaviour is not simply due to chance. Emerging mapping data for 17p13.3 (Stack et al, 1995; White et al, 1996) have suggested two regions: YNZ22 and a more telomeric region (defined by markers D17S926, D17S695, D17S849) may be of interest in human breast cancer. Alongside the HIC-1 (hypermethylated in cancer), ABR and CRK genes (Heisterkamp et al, 1989; Morris et al, 1995; Wales et al, 1995) in this region, 17p13.3 may carry a gene or genes of both scientific interest and clinical importance in breast cancer.

### ACKNOWLEDGEMENTS

The authors thank Dr Wilma Jack, Mrs Ruby Wood and the staff of the Edinburgh Breast Unit for assistance with patient follow-up, Dr A Lessels, Department of Pathology, Western General Hospital, Edinburgh for assistance with the DO1 and DO7 immunohistochemistry. We also thank Professor David Lane, CRC Research Laboratories, University of Dundee, Dundee for the p53 antibodies and Lynne Keir, Department of Surgery, University of Dundee, for secretarial assistance. This work was supported by grants from the Scottish Office Home and Health Department (K/MRS/50/C2153), Scottish Hospitals Endowments Research Trust (Grant 868), Hartwell bequest and Sarah Percy Fund.

### REFERENCES

Aas T, Borresen AL, Geisler S, Smith-Sorensen G, Johnsen H, Varhaug JE, Akslen LA and Lonning PE (1996) Specific P53 mutations are associated with *de novo* resistance to doxorubicin in breast cancer patients. *Nature Med* **2**: 811–814

Allred DC, Clark GM, Elledge R, Fuqua SAW, Brown RW, Chamness GC, Osbourne CK and McGuire WL (1993) Association of p53 protein expression with tumour cell proliferation rate and clinical outcome in node-negative breast cancer. *J Natl Cancer Inst* **85**: 200–206

Andersen TI, Holm R, Nesland JM, Heimdal KR, Ottestad L and Borresen A-L (1993) Prognostic significance of TP53 alterations in breast carcinoma. *Br J Cancer* **68**: 540–548

Barnes DM, Dublin EA, Fisher CJ, Levison DA and Millis RR (1993) Immunohistochemical detection of p53 protein in mammary carcinoma: an important new independent indicator of prognosis? *Hum Pathol* **24**: 469–476

Bergh J, Norberg T, Sjogren S, Lindgren A and Holmberg L (1995) Complete sequencing of the p53 gene provides prognostic information in breast cancer patients, particularly in relation to adjuvant systemic therapy and radiotherapy. *Nature Med* **1**: 1029–1034

Bland KI, Konstadoulakis MM, Vezeridis MP and Wanebo HJ (1995) Oncogene protein co-expression: value of Ha-ras, c-myc, c-fos and p53 as prognostic discriminants for breast carcinoma. *Ann Surg* **221**: 706–720

Borg A, Lennerstrand J, Stenmark-Askmalm M, Ferno M, Brisfors A, Ohrvik A, Stal O, Killander D, Lane DP and Brundell J (1995) Prognostic significance of p53 overexpression in primary breast cancer; a novel luminometric immunoassay applicable on steroid receptor cytosols. *Br J Cancer* **71**: 1013–1017

Borresen A-L, Andersen TI, Eyfjord JE, Cornelis RS, Thorlacius S, Borg A, Johansson U, Theillet C, Scherneck S, Hartman S, Cornelisse C, Hovig E and Devilee P (1995) TP53 mutations and breast cancer prognosis: particularly poor survival rates for cases with mutations in the zinc-binding domains. *Genes Chrom Cancer* **14**: 71–75

Chen LC, Neubauer A, Kurisu W, Waldman FM, Ljung B-M, Goodson W, Goldman ES, Moore D, Balazs M, Liu E, Mayall BH and Smith HS (1991) Loss of heterozygosity on the short arm of chromosome 17 is associated with high proliferative capacity and DNA aneuploidy in primary human breast cancer. *Proc Natl Acad Sci USA* **88**: 3847–3851

Chen TP, Dhingra K, Sahia A, Sneige N, Hortogagyi G and Aldaz CM (1996) Technical approach for the study of the genetic evolution of breast cancer from paraffin-embedded tissue sections. *Br Cancer Res Treat* **39**: 177–185

© Cancer Research Campaign 1998

Cattoretti G, Rilke F, Andreola S, D'Amato L and Delia D (1988) P53 expression in breast cancer. *Int J Cancer* **41**: 178–183

Coles C, Thompson AM, Elder PA, Cohen BB, MacKenzie IM, Cranston G, Chetty U, MacKay J, MacDonald M, Nakamura Y, Hoyheim B and Steel CM (1990) Evidence implicating at least two genes on chromosome 17p in breast carcinogenesis. *Lancet* **336**: 761–763

Cornelis RS, van Vliet M, Vos CBJ, Cleton-Jansen AM, van de Vijver MJ, Peterse JL, Khan PM, Borresen AL, Cornelisse CJ and Devilee P (1994) Evidence for a gene on 17p13.3, distal to TP53, as a target for allele loss in breast tumours without p53 mutations. *Cancer Res* **54**: 4200–4206

Davidoff AM, Herndonn JE, Glover NS, Kerns BJM, Pence JC, Iglehart JD and Marks JR (1991) Relation between p53 overexpression and established prognostic factors in breast cancer. *Surgery* **110**: 259–264

Devilee P, van den Broek M, Kuipers-Dukshoorn N, Kolluri R, Khan PM, Pearson PL and Cornelisse CJ (1989) At least four different chromosomal regions are involved in loss of heterozygosity in human breast carcinoma. *Genomics* **5**: 554–560

Elledge RM and Allred DC (1994) The p53 tumour suppressor gene in breast cancer. *Br Cancer Res Treat* **32**: 39–47

Elledge RM, Clark GM, Fugua SAW, Yu Y and Allred DC (1994) p53 protein accumulation detected by five different antibodies: relationship to prognosis and heat shock protein 70 in breast cancer. *Cancer Res* **54**: 3752–3757

Elledge RM, Gray R, Mansour E, Yu Y, Clark GM, Ravdin P, Osborne CK, Gilchrist K, Davidson NE, Robert N, Tormey DC and Allred DC (1995) Accumulation of p53 protein as a possible predictor of response to adjuvant combination chemotherapy with cyclophosphamide, methotrexate, fluorouracil and prednisone from breast cancer. *J Natl Cancer Inst* **87**: 1254–1256

Friedrichs K, Gluba S, Eidtmann H and Jonat W (1993) Overexpression of p53 and prognosis in breast cancer. *Cancer* **72**: 3641–3647

Hall PA, Meek D and Lane DP (1996) p53 – Integrating the complexity. *J Pathol* **180**: 1–5

Harada Y, Katagiri T, Ito I, Akiyama F, Sakamoto G, Kasumi F, Nakamura Y and Emi M (1994) Genetic studies of 457 breast cancers: clinicopathologic parameters compared with genetic alterations. *Cancer* **74**: 2281–2286

Heisterkamp N, Morris C and Groffen J (1989) ABR, an active BCR-related gene. *Nucleic Acids Res* **17**: 8821–8824

Isola J, Visakorpi T, Holli K and Kallioniemi O-P (1992) Association of overexpression of tumour suppressor protein p53 with rapid cell proliferation and poor prognosis in node-negative breast cancer patients *J Natl Cancer Inst* **84**: 1109–1114

Ito I, Yoshimoto M, Iwase T, Watanabe S, Katagiri T, Harada Y, Kasumi F, Yasuda S, Mitomi T, Emi M and Nakamura Y (1995) Association of genetic alterations on chromosome 17 and loss of hormone receptors in breast cancer. *Br J Cancer* **72**: 438–441

Iwaya K, Tsuda H, Hiraide H, Tamaki K, Tamakuma S, Fukutomi T, Mukai K and Hirohashi S (1991) Nuclear p53 immunoreaction associated with poor prognosis of breast cancer. *Jpn J Cancer Res* **82**: 835–840

Kovach JS, McGovern RM, Cassady JD, Swanson SK, Wold LE, Vogelstein B and Sommer SS (1991) Direct sequencing from touch preparations of human carcinomas: analysis of p53 mutations in breast carcinomas. *J Natl Cancer Inst* **14**: 1004–1009

Lakhani SR, Collins N, Stratton MR and Sloane JP (1995) Atypical ductal hyperplasia of the breast: clonal proliferation with loss of heterozygosity on chromosomes 16q and 17p. *J Clin Pathol* **48**: 611–615

MacGrogan G, Bonichon F, De Mascarel I, Trojani M, Durand M, Avril A and Coindre J-M (1995) Prognostic value of p53 in breast invasive ductal carcinoma: an immunohistochemical study on 942 cases. *Br Cancer Res Treat* **36**: 71–81

Mackay J, Elder PA, Steel CM, Forrest APM and Evans HJ (1988) Allele loss on short arm of chromosome 17 in breast cancers. *Lancet* **ii**: 1384–1385

Marks JR, Humphrey PA, Wu K, Berry D, Bandarenko N, Kerns BJ and Iglehart JD (1994) Overexpression of p53 and HER-2/neu proteins as prognostic markers in early stage breast cancer. *Ann Surg* **219**: 332–341

Martinazzi M, Crivelli F, Zampatti C and Martinazzi S (1993) Relationship between p53 expression and other prognostic factors in human breast carcinoma. An immunohistochemical study. *Am J Clin Pathol* **100**: 213–217

Merlo GR, Venesio T, Bernardi A, Canale L, Gaglia P, Lauro D, Cappa APM, Callahan and Liscia DS (1992) Loss of heterozygosity on chromosome 17p13 in breast carcinomas identifies tumours with high proliferation index. *Am J Pathol* **140**: 215–223

Morris C, Benjes S, Haayaja L, Ledbetter DH, Heisterkamp N and Groffen J (1995) Spatial organisation of ABR and CRK genes on human chromosome band 17p13.3. *Oncogene* **10**: 1009–1011

Munn KE, Walker RA, Mensce L and Varley JM (1996) Mutation of the TP53 gene and allelic imbalance at chromosome 17p13 in ductal carcinoma in situ. *Br J Cancer* **74**: 1578–1585

Nagai MA, Medeiros A, Brentani MM, Brentani RR, Marques LA, Mazoyer S and Mulligan LM (1995) Five distinct deleted regions on chromosome 17 defining different subsets of human primary breast tumours. *Oncology* **52**: 448–453

Osborne RJ, Merlo GR, Mitsudomi T, Venesio T, Liscia DS, Cappa APM, Chiba I, Takahashi T, Nau MM, Callahan R and Minna JD (1991) Mutations in the p53 gene in primary human breast cancers. *Cancer Res* **51**: 6194–6198

Poller DN, Hutchings CE, Galea M, Bell JA, Nicholson RA, Elston CW, Blamey RW and Ellis IO (1992) p53 protein expression in human breast carcinoma: relationship to expression of epidermal growth factor receptor, c-erbB-2 protein overexpression, and oestrogen receptor. *Br J Cancer* **66**: 583–588

Prosser J, Thompson AM, Cranston G and Evans HJ (1990) Evidence that p53 behaves as a tumour suppressor gene in sporadic breast tumours. *Oncogene* **5**: 1573–1579

Radford DM, Fair K, Thompson AM, Ritter JH, Holt M, Steinbrueck T, Wallace M, Wells Jr SA and Donis-Keller HR (1993) Allelic loss on chromosome 17 in ductal carcinoma in situ of the breast. *Cancer Res* **53**: 2947–2950

Runnebaum IB, Nagarajan M, Bowman M, Soto D and Sakumar S (1991) Mutations in p53 as potential molecular markers for human breast cancer. *Proc Natl Acad Sci USA* **88**: 10657–10661

Silvestrini R, Benini E, Daidone MG, Veneroni S, Boracchi P, Cappelletti V, Di Fronzo G and Veronesi U (1993) p53 as an independent prognostic marker in lymph node-negative breast cancer patients. *J Natl Cancer Inst* **85**: 965–970

Silvestrini R, Daidone MG, Benini E, Faranda A, Tomasic G, Boracchi P, Salvadori B and Veronesi U (1996) Validation of p53 accumulation as a predictor of distant metastasis at 10 years of follow-up in 1400 node-negative breast cancers. *Clin Cancer Res* **2**: 2007–2013

Singh S, Simon M, Meybohm I, Jantke I, Jonaf W, Maass H and Goedde HW (1993) Human breast cancer: frequent p53 allele loss and protein overexpression. *Hum Genetics* **90**: 635–640

Stack M, Jones D, White G, Liscia DS, Venesio T, Casey G, Crichton D, Varley J, Mitchell E, Heighway J and Santibanez-Koref M (1995) Detailed mapping and loss of heterozygosity analysis suggest a suppressor locus involved in sporadic breast cancer within a distal region of chromosome band 17p 13.3. *Hum Mol Genet* **4**: 2047–2055

Stenmark-Askmalm M, Stal O, Olsen K, Nordenskjold B and the South East Sweden Breast Cancer Group (1995) p53 as a prognostic factor in stage I breast cancer. *Br J Cancer* **72**: 715–719

Takita K, Sato K, Miyagi M, Watatani M, Akiyama F, Sakamoto G, Kasumi F, Abe R and Nakamura Y (1992) Correlation of loss of alleles on the short arms of chromosomes 11 and 17 with metastasis of primary breast cancer to lymph nodes. *Cancer Res* **52**: 3914–3917

Thompson AM, Steel CM, Chetty U, Hawkins RA, Miller WR, Carter DC, Forrest APM and Evans HJ (1990) p53 gene mRNA expression and chromosome 17p allele loss in breast cancer. *Br J Cancer* **61**: 74–78

Thompson AM, Anderson TJ, Condie A, Prosser J, Chetty U, Carter DC, Evans HJ and Steel CM (1992) p53 allele losses, mutations and expression in breast cancer and their relationship to clinico-pathological parameters. *Int J Cancer* **50**: 528–532

Thorlacius S, Borresen A-L and Eyfjord JE (1993) Somatic p53 mutations in human breast carcinomas in an Icelandic population: a prognostic factor. *Cancer Res* **53**: 1637–1641

Tsuda H, Iwaya K, Fukutomi T and Hirohashi S (1993) p53 mutations and c-erbB-2 amplification in intraductal and invasive breast carcinomas of high histologic grade. *Jpn J Cancer Res* **84**: 394–401

Varley JM, Brammar WJ, Lane DP, Swallow JE, Dolan C and Walker RA (1991) Loss of chromosome 17p13 sequences and mutation of p53 in human breast carcinomas. *Oncogene* **6**: 413–421

Vojtesek B, Bartek J, Midgley CA and Lane DP (1992) An immunochemical analysis of the human nuclear phosphoprotein p53. New monoclonal antibodies and epitope mapping using recombinant p53. *J Immun Meth* **151**: 237–244

Wales MM, Biel MA, El Deiry W, Nelkin BD, Issa J-P, Cavenee WK, Kuerbitz SJ and Baylin SB (1995) p53 activates expression of HIC-1, a new candidate tumour suppressor gene on 17p13.3. *Nature Med* **1**: 570–573

White GRM, Stack M, Santibanezkoref M, Liscia DS, Vensio T, Wang JC, Helms C, Donis-Keller H, Betticher DC, Altermatt HJ, Hoban PR and Heighway J (1996) High levels of loss at the 17p telomere suggest the close proximity of a tumour suppressor. *Br J Cancer* **74**: 863–867

© Cancer Research Campaign 1998